# Salivary pH and Buffering Capacity as Risk Markers for Early Childhood Caries: A Clinical Study

**DOI:** 10.5005/jp-journals-10005-1307

**Published:** 2015-09-11

**Authors:** *D Jayaraj, S Ganesan

**Affiliations:** Assistant Professor, Department of Pedodontics and Preventive Dentistry Mahatma Gandhi Post Graduate Institute of Dental Sciences Gorimedu, Puducherry, India *The author Late Dr D Jayaraj was a mentor and visionary with immense knowledge and experience in the subject. We pay our regards with deepest gratitude to the departed soul to rest in peace.; Associate Professor, Department of Conservative Dentistry and Endodontics Mahatma Gandhi Post Graduate Institute of Dental Sciences Gorimedu, Puducherry, India

**Keywords:** Buffering capacity, Early childhood caries, pH, Risk assessment, Saliva.

## Abstract

**Background:** The diagnostic utility of saliva is currently being explored in various branches of dentistry, remarkably in the field of caries research. This study was aimed to determine if assessment of salivary pH and buffering capacity would serve as reliable tools in risk prediction of early childhood caries (ECC).

**Materials and methods:** Paraffin-stimulated salivary samples were collected from 50 children with ECC (group I) and 50 caries free children (group II). Salivary pH and buffering capacity (by titration with 0.1 N hydrochloric acid) were assessed using a handheld digital pH meter in both groups. The data obtained were subjected to statistical analysis.

**Results:** Statistically, no significant difference was observed between both the groups for all salivary parameters assessed, except for the buffering capacity level at 150 μl titration of 0.1 N hydrochloric acid (p = 0.73; significant at 1% level).

**Conclusion:** Salivary pH and buffering capacity may not serve as reliable markers for risk prediction of ECC.

**How to cite this article:** Jayaraj D, Ganesan S. Salivary pH and Buffering Capacity as Risk Markers for Early Childhood Caries: A Clinical Study. Int J Clin Pediatr Dent 2015;8(3):167-171.

## INTRODUCTION

Early childhood caries (ECC) is defined as the presence of one or more decayed (non-cavitated or cavitated lesions), missing (due to caries), or filled tooth surfaces in any primary tooth in a child 71 months of age or younger. In children younger than 3 years of age, any sign of smooth-surface caries is indicative of severe early childhood caries (S-ECC). From ages 3 to 5 years, one or more cavitated, missing (due to caries), or filled smooth surfaces in primary maxillary anterior teeth; or a decayed, missing, or filled score of ≥ 4 (age 3), ≥ 5 (age 4), or ≥ 6 (age 5) surfaces, constitute S-ECC.^1^

Good oral health is an essential and integral component of good general health. Although enjoying good oral health ensures having more than healthy teeth, many children have inadequate oral and general health because of active and uncontrolled dental caries.^2^ Despite the fact that the prevalence of dental caries has declined over the past decades, ECC remains one of the most common chronic diseases of childhood; especially in developing countries and some minority community in the western world. Yet, little attention and few resources have been spent to understand the nature of this dreadful disease.

The ECC is a virulent form of caries beginning soon after the eruption of primary teeth, develops on smooth surfaces, progressing rapidly, and with a lasting detrimental impact on the dentition. Although the etiology of ECC is similar to that of other types of coronal and smooth surface caries, the biology may differ in some respects. The bacterial flora and host defense systems in the young infant are in the process of being established. In addition, the tooth surfaces are newly erupted and immature, and may show hypoplastic defects.^3^ These unique factors may contribute to the variations in caries susceptibility and risk prediction for ECC, as compared to caries in adults.

It is generally accepted that the caries process is controlled largely by a natural protective mechanism inherent within the saliva. The flow, dilution, pH, buffering, and remineralizing capacity of saliva are recognized as the critical factors that affect, and in some ways, regulate the progression and regression of the caries process. If the oral environment is favorable, saliva can contribute to the strengthening of the tooth by supplying the components known to help and build strong apatite structure. If the oral environment is unfavorable, an adequate flow of saliva can help to dilute and buffer the acid challenge, and thus could slow the rate of damage to the tooth or even repair the damage.^2^

Caries onset and progression is potentially influenced by a diverse group of bacterial, dietary, environmental, socioeconomic and physiological risk factors. Among these, caries experience, the concentrations of mutants-group Streptococci and Lactobacilli, and the buffering capacity of saliva are considered as potential factors for risk assessment.^4^

Despite the well-recognized importance of caries risk assessment, practical models remain yet to be established, especially for children. The role of saliva in the patho-physiological process of ECC remains controversial and unexplored largely. With this background, the present study was aimed to assess the role of salivary pH and buffering capacity in ECC, and to determine if they might be used as potential tools for risk prediction of ECC.

## MATERIALS AND METHODS

Children under the age of 6 years (including those treated by school dental health program) reported to the Department of Pedodontics and Preventive Dentistry, Mahatma Gandhi Post Graduate Institute of Dental Sciences, Puducherry, India, were selected after obtaining informed and written consent from their parents or guardians. The study was approved by the institutional scientific and ethical committee. Children below 6 years fulfilling the criteria for ECC were included under ‘group I’ (study group). Caries free children below 6 years were included in ‘group II’ (control group). Children who were unable to cooperate, or with acute illness, chronic diseases and those under systemic medications were excluded from the study. Simple random sampling technique was followed for selection of subjects. Keeping the power at 80% and p-value at 5% level, the sample size was estimated as 50 children for each of the groups.

A brief history regarding their dietary and oral hygiene practices was obtained. Thorough clinical examination was performed for the assessment of carious status of the children using dmft/dmfs system. Based on the dmft/ dmfs score, their age, and with due considerations for their dietary and oral hygiene practices, the carious status of ‘group I’ children was ranked as mild, moderate or severe form of ECC, as suggested by Wyne et al.^5^

Paraffin-stimulated whole saliva samples collected by direct spitting method were used for the assessment of salivary pH and buffering capacity.^6^ To minimize the diurnal variations in the salivary flow and composition, samples were collected at least 2 hours after meals and at least 1 hour after tooth brushing (once between 10 am and 12 noon or 2 and 4 pm). The children were made to sit straight on the chair and were allowed to relax for few minutes. About 1 gm of unflavored paraffin wax was given to chew for 1 minute and then the initial saliva mixed with wax was asked to spat out. Then, the children were advised to spit the saliva into a graduated container continuously, for a minimum period of 2 minutes. The stimulated salivary quantity (ml) and the flow rate (ml/min) were calculated.^6^

**Fig. 1:**
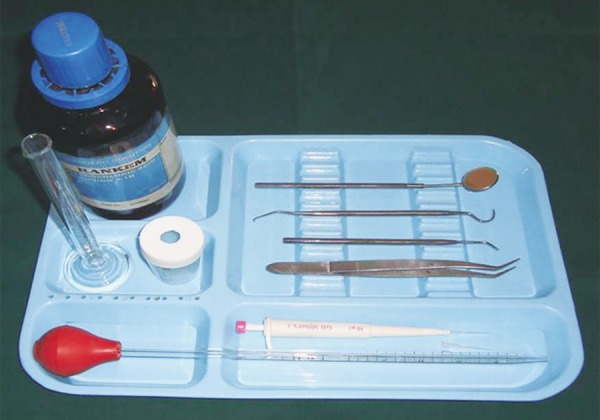
Armamentarium used for caries diagnosis and assessment of salivary pH and buffering capacity

**Fig. 2:**
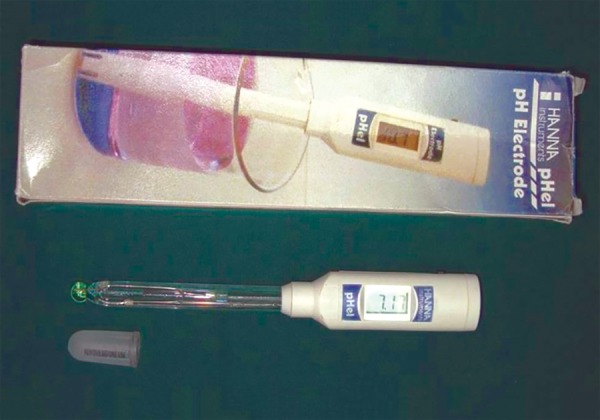
Assessment of salivary pH using digital pH meter

**Fig. 3:**
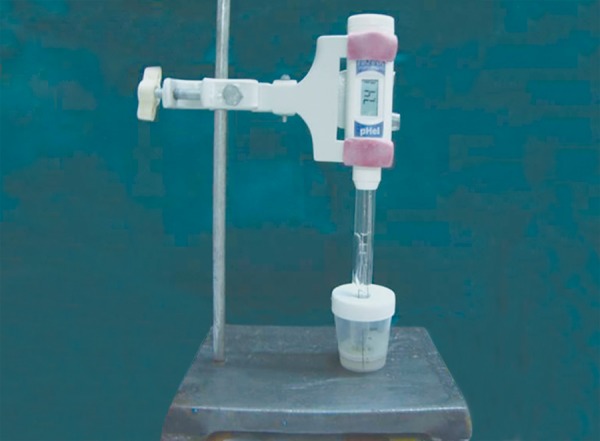
Digital pH meter

Immediately after collection, the pH measurement was done directly, using a hand-held digital pH meter (Hanna, Model pHel 1, Z379395-1EA, Sigma-Aldrich Chemicals Pvt. Ltd, Bengaluru, India) mounted over a sturdy based stand (to avoid variations in the readings due to handling movements) (Figs 1 and 2). The pH meter (dimensions: 200 × 28 × 20 mm; weight: 46 gm) has a long, slim stem with a double junction gel-filled electrode, making it possible to measure little quantity of samples in small vials. The pH meter has a measuring range from 0 to 14 pH and resolution of 0.1 (Fig. 3). The electrode was immersed in the sample in a closed container, the digital reading was allowed to stabilize for few seconds, and the final stable reading was taken as the salivary pH value.

After the assessment of salivary pH, 1 ml of the sample was taken in the closed container and 10 μl of 0.1 N hydrochloric acid (HCl) (Rankem, Ranbaxy, India) was added into it using a micropipette. The sample was then gently shaken to homogenously mix the saliva and the HCl. The pH meter was then immersed into the sample and the stable reading was recorded for salivary buffering capacity. The same procedure was repeated for 50, 100 and 150 μl titrations of 0.1 N HCl; and then the respective readings were recorded for all the samples, to determine the range of salivary buffering capacity.^6^ The salivary buffering capacity value at 50 μl titration of HCl was ranked into one of the following three categories: low buffering capacity (pH < 4.5), medium buffering capacity (pH 4.5-5.5), and high buffering capacity (pH > 5.5); according to the criteria specified by Moritsuka et al.^6^ At periodic intervals, the consistency of pH meter was also monitored, using pH 4 and 7 standard buffers.

The data obtained were tabulated and subjected to appropriate statistical analyses. Unpaired t-test was used to assess the variations between groups I and II children, and for variation between males and females in group I children; F test was used for assessing the variations within the group I children based on their ECC type as mild, moderate and severe.

## RESULTS

A total of 100 children had participated in the study, of which 50 were in ‘group I’ and 50 in ‘group II’. In ‘group I’, 29 were males and 21 females; whereas, in ‘group II’, 22 were males and 28 = females. The mean age for ‘group I’ children was 4.4 years (4.6 and 4.1 years for males and females, respectively), and for ‘group II’ children it was 4 years (4.1 and 3.9 years for males and females, respectively). Out of 50 in group I, five had mild, five had moderate and 40 children had severe ECC.

The mean value of all the salivary parameters for both the groups is summarized in Table 1. In group I, three children had low buffering capacity, seven had medium buffering capacity and 40 had high buffering capacity. In group II, five children had medium buffering capacity and 45 had high buffering capacity.

Unpaired t-test revealed no statistically significant difference (p > 0.05) between the two groups, for all the parameters; except the stimulated salivary buffering capacity level at 150 μl titration of 0.1 N HCl [significant at 1% level (p < 0.01)] (Table 1). Also, there was no significant difference for all these salivary parameters between males and females within group I (Table 2). Comparison within group I based on their ECC type (i.e. between mild, moderate and severe ECC) by F test, also revealed no significant variation (p > 0.05) (Table 3).

## DISCUSSION

The ability to predict an individual’s risk for caries would offer a potentially huge natural way to promote better oral health. Saliva serves as a first line of both non-specific and specific defense in the oral cavity against a number of diseases. Various caries risk assessment models were proposed with salivary analysis as a main component. The present study was aimed at risk prediction for ECC by assessment of salivary pH and buffering capacity. Our study objective was in accordance with the statement by Horowitz, who emphasized the need for research on the effect of salivary constituents on ECC.^7^

**Table 1:** **Table 1:** Comparison of salivary parameters between groups I and II children

		*Group I (control group)* *(n = 50)*		*Group II (study group)* *(n = 50)*					
*Salivary parameters*		*Mean ± SE*		*Mean ± SE*		*t-value*		*p-value*	
Stimulated salivary quantity for 2 minutes		1.78 ± 0.09		1.82 ± 0.12		–0.29		0.77	
Stimulated salivary flow rate		0.89 ± 0.05		0.91 ± 0.06		–0.29		0.77	
Stimulated salivary Ph		6.85 ± 0.05		6.94 ± 0.05		–1.18		0.24	
Buffering capacity at 10 μl HCl titration		6.53 ± 0.04		6.61 ± 0.06		–1.05		0.30	
Buffering capacity at 50 μl HCl titration		6.02 ± 0.06		5.97 ± 0.10		0.35		0.73	
Buffering capacity at 100 μl hci titration		5.22 ± 0.06		5.02 ± 0.11		1.59		0.11	
Buffering capacity at 150 μl HCl titration		4.57 ± 0.06		4.27 ± 0.10		2.58*		0.01	

*p < 0.01 (significant at 1% level)

**Table 2:** **Table 2:** T-test results comparing the salivary parameters between the male and female children in group I

		*Males* * (n = 29)*		*Females* * (n = 21)*					
*Salivary parameters*		*Mean± SE*		*Mean*± *SE*		*t-value*		*p-value*	
Stimulated salivary quantity in 2 minutes		1.72 ± 0.15		1.97 ± 0.19		–1.07		0.29	
Stimulated salivary flow rate		0.86 ± 0.07		0.99 ± 0.09		–1.07		0.29	
Stimulated salivary pH		6.96 ± 0.07		6.91 ± 0.09		0.42		0.67	
Buffering capacity at 10 μl HCl titration		6.62 ± 0.09		6.60 ± 0.08		0.14		0.89	
Buffering capacity at 50 μl HCl titration		6.07 ± 0.12		5.85 ± 0.17		1.10		0.28	
Buffering capacity at 100 μl HCl titration		5.14 ± 0.14		4.85 ± 0.17		1.36		0.18	
Buffering capacity at 150 μl HCl titration		4.33 ± 0.13		4.18 ± 0.15		0.70		0.49	

p > 0.05 for all (not significant)

A total of 100 children had participated in the study, all of which were selected randomly, based on the fulfillments of the specified selection criteria. The children in both the groups were age matched (mean ages were 4.4 and 4 years in groups I and II respectively) and were equal in number. This was done to possibly eliminate changes in the salivary parameters due to the process of development and maturation in children as suggested by Ben Aryeh et al.^8^ The children in both the groups were likely from the same socioeconomic status, with similar feeding and oral hygiene practices that seemed to eliminate the confounding variables between the groups.

Stimulated whole saliva was used for the assessment of salivary pH and buffering capacity, as it was considered the better medium than unstimulated saliva, due to its resistance to variations when subjected to acidic environment.^6^ Salivary pH and buffering capacity were assessed immediately after the sample collection, using a hand-held digital pH meter in a closed container. An assessment in an open environment and delay in time could lead to variations in the value.^9^ The study results revealed no statistically significant difference in the salivary pH and buffering capacity between the two groups and also between males and females within group I children. There was also no significant difference for these parameters within group I children based on their type and severity of ECC.

Analysis of salivary pH and buffering capacity and its correlation with dental caries has given inconsistent results.^9-15^ Lamberts et al studied the salivary pH rise activities in caries free and caries active naval recruits, and found no significant relationship between salivary pH rise activity and caries experience; but, there existed a significant positive correlation between the minimum pH values and bicarbonate content of the samples.^10^

Ericson and Makinen et al^11^ have substantiated an inverse relationship between salivary buffering capacity and caries activity. Gopinath and Arzreanne found that salivary flow rate, viscosity, pH and buffering capacity were lower in subjects with high dental caries.^13^ Our study was in accordance with the study conducted by Schipper et al, Lamberts et al and Tenovuo; but, in conflict with the studies by Ericson, Heintze et al, Gopinath et al and Surdilović et al.^11-15^

Schipper et al stated that the use of saliva as research material might pose particular problems due to its inherent variability and instability.^9^ Salivary flow rate is considered as the most important parameter for cariostatic activity and as such the flow rate have no linear association with dental caries. There seems to exist an individual ‘threshold’ limit which is decisive for enhanced caries activity. This threshold limit varies among different individuals, and therefore, the so-called normal values for unstimulated or stimulated flow rate are more reliable on a population level than among individuals for screening purposes.^15^

The salivary buffering effect has only a weak negative association with caries activity. The decisive processes in caries attack occur within or under the dental plaque, the buffering effect of saliva is limited and obviously more important to screen for erosion than caries-prone individuals. Hence, the assessment of saliva’s functional properties is more important for clinical purposes rather than assessment of individual parameters.^15^

**Table 3:** **Table 3:** F-test results comparing the salivary parameters across the group I children by early childhood caries type

		*Early childhood caries type*									
		*Mild* *(n = 5)*		*Moderate* *(n = 5)*		*Severe* *(n = 40)*					
*Salivary parameters*		*Mean* ± *SE*		*Mean*± *SE*		*Mean* ± *SE*		*f-value*		*p-value*	
Stimulated salivary quantity in 2 minutes		2.20 ± 0.46		1.44 ± 0.21		1.83 ± 0.13		1.05		0.36	
Stimulated salivary flow rate		1.10 ± 0.23		0.72 ± 0.11		0.91 ± 0.07		1.05		0.36	
Stimulated salivary pH		6.99 ± 0.24		6.70 ± 0.19		6.96 ± 0.06		1.08		0.35	
Buffering capacity at 10 μl HCl titration		6.66 ± 0.20		6.43 ± 0.21		6.63 ± 0.07		0.47		0.63	
Buffering capacity at 50 μl HCl titration		6.21 ± 0.32		5.88 ± 0.17		5.96 ± 0.12		0.32		0.72	
Buffering capacity at 100 μl HCl titration		5.39 ± 0.32		4.91 ± 0.24		4.99 ± 0.13		0.67		0.52	
Buffering capacity at 150 μl HCl titration		4.72 ± 0.22		4.36 ± 0.30		4.20 ± 0.11		1.30		0.28	

p > 0.05 for all (f-values are not significant)

Although both the salivary secretion (flow) rate and buffering pH (buffering capacity) are related to dental caries, neither of them when used singly showed a sufficient correlation to caries activity of an individual. When these parameters were used in combinations with several other indications of increased risk for caries *(Streptococcus mutants,* Lactobacilli, diet, drugs, medical disorders, etc.), they form useful tools in the diagnosis of the potential caries activity or prediction of the risk for dental caries in an individual.^16^

Also, consideration in children for variations due to the natural developmental process is vital while utilizing salivary parameters for caries risk prediction. Our study revealed that salivary pH and buffering capacity assessment alone does not serve as reliable tools for ECC risk prediction. Hence, further research that explores the functional properties of whole saliva as well as the role of its individual components, with appropriate considerations for age, may serve as better caries risk assessment models.

## CONCLUSION

No significant difference in the salivary pH and buffering capacity between caries free children and children with ECC was observed. Thus, the assessment of salivary pH and buffering capacity alone may not serve as reliable tools for risk prediction of ECC.
